# QTL mapping and whole-genome sequencing analysis for novel genetic resources associated with sucrose content in soybean [*Glycine max* (L.) Merr.]

**DOI:** 10.1007/s00122-024-04808-5

**Published:** 2025-02-03

**Authors:** Dongho Lee, Tri D. Vuong, James G. Shannon, Qijian Song, Feng Lin, Henry T. Nguyen

**Affiliations:** 1 Fisher Delta Research, Extension, and Education Center, Division of Plant Science and Technology, University of Missouri, Portageville, MO 63873 USA; 2 Division of Plant Science and Technology, University of Missouri, Columbia, MO 65211 USA; 3 Present Address: Soybean Genomics and Improvement Laboratory, Beltsville Agricultural Research Center, USDA-ARS, Beltsville, MD 20705 USA

## Abstract

**Key message:**

A major QTL for sucrose content was mapped on chromosome 8 in PI 506593. The novel genetic variants and candidate genes were further identified within the major QTL.

**Abstract:**

Sucrose in soybean [*Glycine max* (L.) Merr.] contribute to animal feed efficiency and natural sweetness of soy products. Thus, identifying novel genetic resources, such as quantitative trait loci (QTL), associated with sucrose content in soybean is essential for enhancing seed values. In this study, two recombinant inbred line populations derived from the same high sucrose donor parent, PI 506593, were used to identify significant QTLs. A total of 11 sucrose-related regions on chromosomes (Chrs.) 4, 5, 6, 8, 10, and 13 were identified using QTL analysis. Among them, four QTLs (*qSUC_08.1*, *qSUC_08.2*, *qSUC_08.3*, and *qSUC_08.4*) were clustered in the interval of 40,597,410–42,861,364 bp on Chr. 8, which was considered major QTL region. A desirable marker at 41,834,095 bp was tested in two populations, showing that two phenotypically extreme groups were efficiently differentiated. We further identified 44 and 54 candidate genes with non-synonymous mutations in the major QTL region based on the annotations of Wm82.a2.v1 and Wm82.a5.v1 assemblies, respectively. Among 54 candidate genes from Wm82.a5.v1, Protein Variation Effect Analyzer (PROVEAN) revealed that 18 genes contained 34 variants that had deleterious impacts on biological functions. RNA-seq analysis highlighted five candidate genes that were highly expressed in pod and seed tissues during reproductive stages and other plant parts. A gene, *Gm_Wm82_23219* (*Glyma.08G293800*, Wm82.a2.v1) encoding proline-rich protein 4-like, was highlighted in both PROVEAN and RNA-seq analyses. Novel findings in this study will be valuable genetic resources in soybean breeding programs that aim to improve efficiency in animal feed and human food.

**Supplementary Information:**

The online version contains supplementary material available at 10.1007/s00122-024-04808-5.

## Introduction

Soybean [*Glycine max* (L.) Merr.] is the leading protein meal source, accounting for 69.7% (247 million metric tons) of global protein meal consumption, followed by rapeseed (*Brassica napus* L.) (13.1%, 47 million metric tons) in 2023 (http://soystats.com). Soybean meal is primarily used to feed non-ruminant animals, such as poultry and swine, as a main source of high-quality protein in feed formulations (Pope et al. 2023). Soybean meal meets three fundamental requirements in animal feeding programs, which are (1) Having one or more essential nutrients, (2) Being available to meet the demand of regular usage on a large scale, and (3) Being cost-effective to use (Dozier and Hess 2011). Besides protein, soluble carbohydrates are also essential components in animal feed for improving metabolizable energy (ME) efficiency. Soybean seeds generally contain up to 15% soluble carbohydrates on a dry-weight basis (Feng et al. 2005). The three main components consisting of soluble carbohydrates are sucrose, raffinose, and stachyose, of which sucrose typically accounts for the largest portion of soluble carbohydrates in cultivated soybean seeds (Sui et al. 2020; Wang et al. 2023a).

Among soluble carbohydrates in soybean, sucrose is the only nutritionally beneficial component, which is readily digestible by non-ruminant animals and turns into the source of ME (Jo et al. 2018; John 2008; Parsons et al. 2000; Wang et al. 2023a). Animal feeding programs recommend feed formulations with high sucrose content because sucrose has a significantly higher ME value (3900 kcal/kg) than starch (2918–3396 kcal/kg) (John 2008; Ostezan et al. 2023). Additionally, as the global vegetarian population has increased in recent decades, soy products have garnered popularity as a substitute for meat protein in vegetarian diets (Simeone et al. 2022; Wang et al. 2023b). Sucrose is a pivotal contributor to natural sweetness in soy food products, such as edamame, miso, natto, tofu, and soymilk (Rosset et al. 2012; Sui et al. 2020; Wang et al. 2023a). Therefore, the development of high sucrose soybean varieties has become a goal of breeding programs to improve animal feed efficiency and natural sweetness to meet consumer preference for soy products.

Quantitative trait loci (QTL) mapping strategies have been mainly used to identify genomic regions governing complex quantitative traits (Rani et al. 2023). For sucrose content in soybean seed, Maughan et al. (2000) first reported 17 sucrose-related QTLs on chromosomes (Chrs.) 5, 7, 8, 13, 15, 19, and 20 using an interspecific cross between a large-seeded breeding line and a wild soybean (*G. soja*) line. Kim et al. (2005, 2006) identified 17 sucrose-related QTLs on Chrs. 2, 11, 12, 14, 15, 16, and 19 using two F_2:10_ recombinant inbred line (RIL) populations sharing the same high sucrose donor line, Keunolkong. A major QTL for sucrose content was identified on Chr. 11 in V99-5089, derived from a cross between V71-370 and PI 87013 (Saghai Maroof and Buss 2008). Skoneczka et al. (2009) used two F_2:3_ populations derived from PI 200508 to identify another major QTL on Chr. 6 for high sucrose content. Later, three sucrose-related QTLs on Chrs. 7, 11, and 20 were identified by Wang et al. (2014), of which the QTL on Chr. 11 was the same QTL that Saghai Maroof and Buss (2008) previously identified. Zeng et al. (2014) identified three sucrose-related QTLs on Chrs. 5, 9, and 16. There have been more QTL mapping studies conducted for sucrose content in soybean using different donor parents and larger marker data sets (Akond et al. 2015; Knizia et al. 2023; Lee et al. 2016; Liu et al. 2023; Patil et al. 2018; Salari et al. 2021).

However, only a few variants and genes have been identified to be associated with high sucrose content. A three-base pair deletion leading to an amino acid deletion (*rs2W331-*) and a missense mutation (*rs2T107I*) in raffinose synthase 2 (RS2, *Glyma.06G179200*, Wm82.a2.v1) coding regions were responsible for increased sucrose and reduced raffinose and stachyose content (Dierking and Bilyeu 2008, 2009; Kerr and Sebastian 2000; Skoneczka et al. 2009). Although mutations in RS3 (*Glyma.05G003900*, Wm82.a2.v1) (*rs3snp5*/*rs3snp6*) alone did not increase the sucrose content, the combination of the RS2 and RS3 mutations significantly increases sucrose content (Hagely et al. 2020; Jo et al. 2018; Lee et al. 2023a; Thapa et al. 2019). A natural mutation in the D-myo-inositol 3-phosphate synthase 1 (MIPS1) gene (*Glyma.11G238800*, Wm82.a2.v1) derived from V99-5089 was associated with elevated sucrose content and significantly reduced raffinose and stachyose content in soybean (Rosso et al. 2011; Saghai Maroof and Buss 2008).

Recently, a new high-quality reference genome of Williams 82 (Wm82.a5.v1) was assembled using the combination of PacBio HiFi sequencing and Bionano optical mapping (Garg et al. 2023). The Wm82.a5.v1 conveys much smaller gaps and scaffold breaks than previous versions, such as Wm82.a1.v1.1 (Schmutz et al. 2010), Wm82.a2.v1 (Song et al. 2016), and Wm82.a4.v1 (Valliyodan et al. 2019). The previous reference genome assemblies contained 56,691 gaps (42.29 Mbp), whereas Wm82.a5.v1 is near gapless (only 14.25 Kbp gaps across the genome), of which ten chromosomes were completely reconstructed without any gaps (Garg et al. 2023). The high-quality reference genome recently developed will provide a robust and accurate understanding of the genetic architecture underlying complex quantitative traits of interest.

In this study, a high sucrose soybean germplasm, PI 506593, was crossed with two low sucrose breeding lines, S16-14161 and S16-11651, to develop two RIL mapping populations. The populations were genotyped with BARCSoySNP6K Illumina Infinium BeadChips (Song et al. 2020) and phenotypically evaluated in three field environments to identify sucrose-related QTLs. Furthermore, we compared the whole-genome sequence (WGS) of the three parental lines after alignment with the most widely used soybean reference genome assembly (Wm82.a2.v1) and the new soybean reference genome assembly (Wm82.a5.v1) to identify candidate genes with novel SNP and Indel variants within the major QTL in PI 506593. Protein Variation Effect Analyzer (PROVEAN) and RNA-seq analysis were further used to investigate the biological function of candidate genes, the impact of novel variants, and the gene expression levels of candidate genes across tissues and developmental stages. This study provides valuable information on QTL and candidate genes controlling sucrose content in soybean.

## Materials and methods

### Plant materials

PI 506593 was selected from the genome-wide association study panel used by Lee et al. (2023b) based on the sucrose content and stability across multiple environments. PI 506593 is a high sucrose large-seeded (32 g 100 seeds^−1^) maturity group VI soybean germplasm originating from Japan (https://www.ars-grin.gov/). Two low sucrose breeding lines, S16-14161 and S16-11651 (Chen et al. 2022), developed by the University of Missouri—Fisher Delta Research, Extension, and Education Center (FDREEC), were crossed with PI 506593.

The crosses of S16-14161 × PI 506593 and S16-11651 × PI 506593 were made in the summer of 2019 at the FDREEC in Portageville, MO, USA. The F_1_ and F_2_ generations were grown in the East Campus Plant Growth Facility at the University of Missouri, Columbia, MO, USA, and F_3_ seeds were harvested in October 2020. The F_3_ and F_4_ generations were grown in the soybean winter nursery in Costa Rica until mid-June 2021. The modified single-pod descent method was used throughout the generation advancement process (Fehr 1987). A total of 140 and 207 F_4:5_ RILs constituted two mapping populations, named P593_RIL1 (S16-14161 × PI 506593) and P593_RIL2 (S16-11651 × PI 506593), respectively, and were subsequently used for QTL analysis.

### Field experiments

The two RIL populations and the parental lines were planted in three field environments. It included the FDREEC, Portageville, MO, USA (36.42°N 89.70°W) in 2021 and 2022 (named FDREEC_21 and FDREEC_22, respectively) and the Bradford Research and Extension Center (BREC), Columbia, MO, USA (38.89°N 92.19°W) in 2022 (named BREC_22). Ten seeds of each line were planted in 75 cm wide rows in hill plots spaced 30 cm apart at the FDREEC and 60 cm apart at the BREC in a randomized complete block design (RCBD) with two replications. Seeds were harvested at maturity for further sucrose quantification.

### Phenotyping for sucrose content

The F_5:6_ and F_6:7_ RIL populations harvested in 2021 and 2022, respectively, were phenotyped for sucrose content. Ten intact soybean seeds (fully matured, spotless, and no damage) were sampled from each plot and used to quantify sucrose content using the established High-Performance Liquid Chromatography (HPLC) protocol described by Valliyodan et al. (2015) with minor modifications. Briefly, a powder sample was obtained by grinding seed samples with Thomas Wiley Mini-Mill (Arthur Thomas Co., Chadds Ford, PA, USA) fitted with a 20-mesh screen. The samples were lyophilized for two days using a Labconco freeze-dry system (Labconco, Kansas City, MO, USA). HPLC-grade water of 900 μL was added to 90.25 (± 0.15) mg of each lyophilized sample in a 2 mL centrifuge tube. After incubating tubes at 55 °C with 200 rpm agitation for an hour, the tubes were vortexed for 30 s. After 20 min under room temperature, 900 μL HPLC-grade acetonitrile was added to each tube. Then, the suspension was centrifuged for 30 min at a 14.0 × 1000 min^−1^ × g speed. The supernatant was diluted five times using 65% HPLC-grade acetonitrile to prepare the final samples. The Agilent HPLC-ELSD (Evaporative Light Scattering Detection) 120 series (Agilent, Santa Clara, CA, USA), equipped with the Prevail Carbohydrate ES columns (5 μm 250 × 4.6 mm) and guard columns (7.5 × 4.6 mm) (Grace Davison Discovery Sciences, Deerfield, IL, USA) were used to quantify sucrose content. The calibration curves were generated using the standard mixtures prepared in HPLC-grade water with 50, 100, 300, 500, and 1000 μg/mL concentrations.

### Genotypic data

The genomic DNA of the F_5:6_ RIL populations and parental lines were isolated from the youngest trifoliate leaves using an established cetyltrimethylammonium bromide (CTAB) protocol with minor modifications as previously described (Vuong et al. 2010). Marker genotyping was performed in the Soybean Genomics and Improvement Laboratory, USDA-ARS, Beltsville, MD, using the BARCSoySNP6K Illumina Infinium BeadChips (Illumina Inc., San Diego, CA, USA) (Song et al. 2020). The SNP alleles were called using the Genotyping Module 2.0 in GenomeStudio software (https://www.illumina.com/). The physical positions of SNP markers were aligned to Wm82.a2.v1. The WGS data of parental lines were generated using the Illumina NovaSeq PE150 platform at a depth of 30 × by Novogene Corporation Inc. (Sacramento, CA, USA). Sequencing libraries with an insert size of ~ 350 bp were constructed incorporating the standard protocol.

### Variant identification

The WGS data of the three parental lines, S16-14161, S16-11651, and PI 506593, were further used for variant identification. The raw Illumina reads were trimmed using Trimmomatic version 0.39 (Bolger et al. 2014). The clean data were aligned to two reference genome assemblies, Wm82.a2.v1 and Wm82.a5.v1, respectively, using the BWA-MEM (Li and Durbin 2009). The genome assemblies and annotation files of Wm82.a2.v1 and Wm82.a5.v1 were obtained from Soybase (https://www.soybase.org/) and Garg et al. (2023), respectively. The entire variant calling processing followed the industry-standard pipeline in GATK (available at https://gatk.broadinstitute.org/hc/en-us). The variants were annotated using the ANNOVAR tool (Wang et al. 2010). The physical positions of QTL regions and genes on Wm82.a5.v1 were retrieved from a liftover file with corresponding gene IDs and physical positions between Wm82.a2.v1 and Wm82.a5.v1. The genes containing unique variants in a donor parent were further evaluated.

### Genetic map construction and QTL analysis

A genetic linkage map was constructed with a 6K SNP dataset for each population using JoinMap version 5.0 software (Van Ooijen 2018). A logarithm of the odds (LOD) score from 2 to 15 was used to cluster linkage groups. Regression mapping was set for the mapping algorithm, and map distances were calculated using Kosambi’s mapping function. The QTL analysis was carried out using MapQTL version 6.0 software based on a genetic linkage map, SNP alleles, and phenotypic data (Van Ooijen 2009). Successive procedures, including interval mapping, automatic cofactor selection, and multi-QTL method, were performed for QTL analysis. The genome-wide LOD threshold was determined using a 1000-permutation test at the 0.05 probability level of significance, resulting in the LOD threshold of 3.2 being selected for both populations in this study. The significant QTL region was defined by two flanking markers encompassing the marker with a LOD score higher than 3.2. The QTL identified in this study were named according to the following rule: *qSUC*_chromosome.QTL number (e.g., *qSUC_08.1*, *qSUC_08.2*, etc.).

### Candidate gene identification

First, all genes within the QTL region on Wm82.a2.v1 and Wm82.a5.v1 were respectively retrieved. Afterward, candidate genes with non-synonymous SNP and Indel variants present only in PI 506593 were collected based on the variant identification results on Wm82.a2.v1 and Wm82.a5.v1. The functional annotations of those candidate genes were studied based on Wm82.a5.v1 using Soybase (https://www.soybase.org/). For unannotated candidate genes, coding sequences were used to perform BLAST in NCBI (https://blast.ncbi.nlm.nih.gov/Blast.cgi) based on soybean (*Glycine max*), wild soybean (*Glycine soja*), common bean (*Phaseolus vulgaris*), and Arabidopsis (*Arabidopsis thaliana*) genome databases. The biological impacts of amino acid changes derived from non-synonymous SNP and non-frameshift Indel variants were predicted by PROVEAN version 1.1.5 software (Choi and Chan 2015). The amino acid sequences used for PROVEAN were based on Wm82.a5.v1, and the significant threshold of ≤ − 2.5 was used, as suggested by Choi and Chan (2015). RNA-seq data analysis was conducted based on two gene expression data sets publicly available from Severin et al. (2010) and Almeida-Silva et al. (2023), respectively, as described in Lee et al. (2023b). The PROVEAN and RNA-seq analysis were conducted for the candidate genes identified in Wm82.a5.v1.

### Statistical analysis

The broad-sense heritability (*H*^2^) was calculated using the *H2cal* function in the *inti* R package (https://rdrr.io/cran/inti/src/R/H2cal.R). Analysis of variance (ANOVA) was conducted using the PROC MIXED procedure of SAS software version 9.4 (SAS Institute, Cary, NC, USA). Genotype was used as a fixed effect, and Environment, Genotype × Environment interaction, and replication were used as random effects.

## Results

### Phenotypic variations of sucrose content in two RIL populations

The overall description of the sucrose content of two RIL populations (P593_RIL1 and P593_RIL2) across three environments is shown in Table 1. In P593_RIL1, there was transgressive segregation for sucrose content with a normal distribution (Shapiro–Wilk value from 0.988 to 0.992) in all environments (Table 1). PI 506593 consistently showed higher sucrose content (8.2%) than S16-14161 (5.5%) across environments. P593_RIL1 had the highest average sucrose content in BREC_22 (8.3%) and the lowest in FDREEC_21 (6.9%). The coefficient of variation (CV) ranged from 10.4% to 12.9%, and the broad-sense heritability was 0.83. Similarly, the sucrose content of P593_RIL2 showed normal distribution (Shapiro–Wilk value from 0.975 to 0.996) and transgressive segregation in all environments (Table 1). PI 506593 consistently showed higher sucrose content (8.2%) than S16-11651 (6.5%) in P593_RIL2 across environments. The sucrose content of P593_RIL2 also varied across environments, following a similar trend as P593_RIL1, with the highest average sucrose content observed in BREC_22 (7.8%) and the lowest in FDREEC_21 (6.6%). The CV ranged from 10.0 to 12.6%, and the broad-sense heritability was 0.84. The ANOVA showed that except for the genotype × environment interaction in P593_RIL1, all other factors had significant effects (Suppl. Table S1).

**Table 1:** The overall description of sucrose content in parents (P1 and P2) and two mapping populations across three environments

Population^a^	Env^b^	P1	P2	Mean ± SD^c^	Range	Shapiro wilk (w)	Skewness	Kurtosis	Variance	CV (%)^d^	Heritability (*H*^*2*^)^e^
P593_RIL1	FDREEC_21	4.9	7.8	6.9 ± 0.9	4.5–9.4	0.991	− 0.01	3.02	0.89	12.9	0.83
	FDREEC_22	5.4	8.0	7.2 ± 0.9	5.3–10.1	0.988	0.30	3.34	0.75	10.4	
	BREC_22	6.3	8.8	8.3 ± 1.0	5.8–10.8	0.992	0.15	3.00	0.93	11.2	
	Mean	5.5	8.2	7.4 ± 0.8	5.1–9.4	–					
P593_RIL2	FDREEC_21	5.9	7.8	6.6 ± 0.9	4.4–9.1	0.990	0.07	2.61	0.83	12.6	0.84
	FDREEC_22	6.5	8.0	6.9 ± 0.9	3.8–9.5	0.996	− 0.05	3.24	0.79	11.4	
	BREC_22	7.0	8.8	7.8 ± 0.9	6.1–10.8	0.975	0.54	3.10	0.78	10.0	
	Mean	6.5	8.2	7.1 ± 0.8	5.2–9.0	–					

^a^ P593_RIL1, a bi-parental mapping population from a cross between S16-14161 (P1) and PI 506593 (P2); P593_RIL2, a bi-parental mapping population from a cross between S16-11651 (P1) and PI 506593 (P2)

^b^ FDREEC_21, Fisher Delta Research, Extension, and Education Center in 2021; FDREEC_22, Fisher Delta Research, Extension, and Education Center in 2022; BREC_22, Bradford Research Education Center in 2022

^c^ SD, standard deviation

^d^ CV, coefficient of variation

^e^ *H*^*2*^, broad sense of heritability

### Genetic linkage map for P593_RIL1 and P593_RIL2

Among 6,000 markers from the BARCSoySNP6K BeadChips, 1440 (24%) and 600 (10%) markers were polymorphic between the two parents of the P593_RIL1 (S16-14161 × PI 506593) and P593_RIL2 (S16-11651 × PI 506593), respectively (Table 2). The polymorphic markers were anchored on 20 chromosomes of the integrated genetic map with a total genetic distance of 2422.4 cM in P593_RIL1 and 2695.2 cM in P593_RIL2. In P593_RIL1, the total distance of each chromosome ranged from 74.9 cM (Chr. 7) to 187.5 cM (Chr. 13), and the genetic interval between two neighboring markers averaged from 1.3 to 2.6 cM, with an average of 1.7 cM. In P593_RIL2, the total distance of each chromosome ranged from 54.2 cM (Chr. 7) to 217.1 cM (Chr. 2), and the average genetic interval between two neighboring markers varied from 2.0 to 15.5 cM, with an average of 5.2 cM.

**Table 2:** Distribution of SNP markers across 20 chromosomes

Chr^a^	P593_RIL1^b^			P593_RIL2^c^		
	# Markers	Total distance (cM)	Average interval (cM)	# Markers	Total distance (cM)	Average interval (cM)
1	48	114.8	2.4	21	176.1	8.4
2	99	163.2	1.6	14	217.1	15.5
3	74	113.1	1.5	35	81.7	2.3
4	84	131.3	1.6	22	159.4	7.2
5	56	123.3	2.2	18	120.2	6.7
6	75	159.7	2.1	23	115.4	5.0
7	57	74.9	1.3	27	54.2	2.0
8	104	141.4	1.4	24	118.5	4.9
9	79	117.6	1.5	31	187.2	6.0
10	80	143.2	1.8	28	127.4	4.6
11	57	146.2	2.6	23	93.0	4.0
12	67	97.8	1.5	18	124.1	6.9
13	82	187.5	2.3	61	176.4	2.9
14	62	82.0	1.3	51	133.1	2.6
15	62	107.3	1.7	16	81.0	5.1
16	69	101.2	1.5	39	189.2	4.9
17	83	121.3	1.5	44	102.6	2.3
18	59	75.8	1.3	37	130.0	3.5
19	74	115.6	1.6	29	125.5	4.3
20	69	105.2	1.5	39	183.1	4.7
**Total**	**1440**	**2422.4**	**1.7**	**600**	**2695.2**	**5.2**

^a^ Chr, chromosome

^b^ P593_RIL1, a bi-parental mapping population from a cross between S16-14161 and PI 506593

^c^ P593_RIL2, a bi-parental mapping population from a cross between S16-11651 and PI 506593

### Identification of QTLs associated with sucrose content

The QTL analysis of the two mapping populations identified 11 sucrose-related QTLs on Chrs. 4, 5, 6, 8, 10, and 13 (Table 3). In P593_RIL1, six QTLs were identified on Chrs. 6 (*qSUC_06.1* and *qSUC_06.2*), 8 (*qSUC_08.1* and *qSUC_08.2*), 10 (*qSUC_10.1*), and 13 (*qSUC_13.1*) and explained 9.1–14.9% of the phenotypic variation (Table 3). Among them, *qSUC_08.1* and *qSUC_06.1* had the highest LOD values of 5.9 and 5.3 in the FDREEC_21 and FDREEC_22, respectively. Only one QTL (*qSUC_06.2*) was detected with a LOD value of 4.1 in BREC_22. The additive effects of each QTL ranged from − 0.4 to -0.3, indicating that high sucrose-related alleles were inherited from the donor parent, PI 506593.

**Table 3:** Summary of QTL associated with sucrose content in two mapping populations across three environments

Population^a^	Environment	QTL^b^	Chr^c^	Marker interval (cM)	Physical interval (bp)	LOD^d^	PVE (%)^e^	Add^f^
P593_RIL1	FDREEC_21	*qSUC_08.1*	8	112.9–114.8	42,146,252–42,532,915	5.9	14.0	− 0.4
	FDREEC_21	*qSUC_10.1*	10	49.9–51.5	37,502,435–37,995,354	4.5	10.4	− 0.3
	FDREEC_21	*qSUC_13.1*	13	71.5–76.7	25,268,844–25,927,261	4.0	9.3	− 0.3
	FDREEC_22	*qSUC_06.1*	6	115.2–124.0	16,133,204–21,786,787	5.3	14.9	− 0.3
	FDREEC_22	*qSUC_08.2*	8	114.8–115.9	42,532,915–42,861,364	3.3	9.1	− 0.3
	BREC_22	*qSUC_06.2*	6	124.0–131.4	21,786,787–46,805,251	4.1	13.9	− 0.4
P593_RIL2	FDREEC_21	*qSUC_05.1*	5	77.5–86.1	34,272,637–35,101,291	5.2	7.8	− 0.3
	FDREEC_21	*qSUC_08.3*	8	100.8–103.2	40,940,261–41,834,095	12.1	19.7	− 0.4
	FDREEC_21	*qSUC_13.2*	13	116.9–117.1	32,525,645–32,570,888	3.7	5.5	− 0.2
	FDREEC_22	*qSUC_08.4*	8	99.7–100.8	40,597,410–40,940,261	5.3	11.3	− 0.3
	BREC_22	*qSUC_04.1*	4	106.0–125.4	46,793,849–48,002,103	3.4	6.3	− 0.2
	BREC_22	*qSUC_08.4*	8	99.7–100.8	40,597,410–40,940,261	3.8	7.1	− 0.3

^a^ P593_RIL1, a bi-parental mapping population from a cross between S16-14161 and PI 506593; P593_RIL2, a bi-parental mapping population from a cross between S16-11651 and PI 506593

^b^ QTL, quantitative trait loci

^c^ Chr, chromosome

^d^ LOD, logarithm of the odds

^e^ PVE, percentage variance explained

^f^ Add, additive effect

In P593_RIL2, five QTLs were identified on Chrs. 4 (*qSUC_04.1*), 5 (*qSUC_05.1*), 8 (*qSUC_08.3* and *qSUC_08.4*), and 13 (*qSUC_13.2*) and explained 5.5–19.7% of the phenotypic variation (Table 3). Among them, *qSUC_08.3* and *qSUC_08.4* showed the highest LOD values of 12.1 and 3.8 in FDREEC_21 and BREC_22, respectively, while *qSUC_08.4* was the only QTL detected in FDREEC_22 with a LOD value of 5.3. Similarly, in P593_RIL1, the additive effects of each QTL varied from − 0.4 to − 0.2, indicating that PI 506593 positively contributed to an increase in sucrose content. The QTL analysis of two populations consistently pinpointed a QTL region on Chr. 8 harboring four QTLs (*qSUC_08.1*, *qSUC_08.2*, *qSUC_08.3*, and *qSUC_08.4*) at the physical interval of 40,597,410–42,861,364 bp across all environments (except BREC_22 in the P593_RIL1) (Table 3). This region should be considered the location of a major QTL conferring high sucrose content in PI 506593 for further investigation.

### Sucrose content of selected RILs in each population based on the desirable SNP marker

Within the major QTL region on Chr. 8, the most desirable SNP marker was selected based on the correlation with the sucrose content of RILs in each population. The SNP marker at 41,834,095 bp on Chr. 8 was selected for both populations. Fifteen RILs from each phenotypically extreme group (Top vs. Bottom) were selected from both populations, and their sucrose content and alleles of the SNP marker were described in Table 4. In P593_RIL1, the top 15 RILs showed average sucrose content ranging from 8.8 to 10.2%, and 12 RILs carried an allele derived from the donor parent. On the other hand, the bottom 15 RILs showed average sucrose content from 5.2 to 6.3%, and 13 RILs carried an allele derived from the low sucrose parental line. In P593_RIL2, the top 15 RILs showed average sucrose content ranging from 8.2 to 9.0%, but only six RILs carried an allele derived from the donor parent. However, all the bottom 15 RILs carried an allele derived from the low sucrose parent, with average sucrose content ranging from 5.2 to 6.0%. Overall, the desirable SNP marker demonstrated high efficiency in differentiating between two extreme groups, while it was only efficient in discriminating the low sucrose group in P593_RIL2.

**Table 4:** Sucrose content of the selected lines from extreme groups based on the desirable SNP marker within the P593_RIL1 and P593_RIL2, respectively

Population^a^	Top 15 RILs			Bottom 15 RILs		
	RIL ID	Allele^b^	Sucrose (%)^c^	RIL ID	Allele	Sucrose (%)
P593_RIL1	P593_RIL1_96	B	10.2	P593_RIL1_114	B	6.3
	P593_RIL1_177	B	9.8	P593_RIL1_181	B	6.3
	P593_RIL1_192	A	9.5	P593_RIL1_102	B	6.2
	P593_RIL1_35	A	9.4	P593_RIL1_152	B	6.1
	P593_RIL1_101	A	9.4	P593_RIL1_13	B	6.1
	P593_RIL1_60	B	9.4	P593_RIL1_138	B	6.1
	P593_RIL1_54	A	9.3	P593_RIL1_189	A	6.0
	P593_RIL1_196	A	9.2	P593_RIL1_180	A	5.9
	P593_RIL1_173	A	9.1	P593_RIL1_24	B	5.8
	P593_RIL1_48	A	9.1	P593_RIL1_153	B	5.7
	P593_RIL1_28	A	9.0	P593_RIL1_52	B	5.7
	P593_RIL1_105	A	9.0	P593_RIL1_17	B	5.5
	P593_RIL1_120	A	9.0	P593_RIL1_15	B	5.4
	P593_RIL1_38	A	8.8	P593_RIL1_89	B	5.2
	P593_RIL1_61	A	8.8	P593_RIL1_155	B	5.2
P593_RIL2	P593_RIL2_88	B	9.0	P593_RIL2_114	B	6.0
	P593_RIL2_100	B	9.0	P593_RIL2_116	B	6.0
	P593_RIL2_47	A	8.8	P593_RIL2_261	B	6.0
	P593_RIL2_27	A	8.8	P593_RIL2_300	B	6.0
	P593_RIL2_212	B	8.7	P593_RIL2_269	B	5.9
	P593_RIL2_76	B	8.7	P593_RIL2_117	B	5.9
	P593_RIL2_254	A	8.6	P593_RIL2_63	B	5.8
	P593_RIL2_146	A	8.5	P593_RIL2_164	B	5.8
	P593_RIL2_3	A	8.4	P593_RIL2_215	B	5.8
	P593_RIL2_220	B	8.4	P593_RIL2_178	B	5.7
	P593_RIL2_256	B	8.3	P593_RIL2_128	B	5.7
	P593_RIL2_240	A	8.3	P593_RIL2_305	B	5.5
	P593_RIL2_202	B	8.3	P593_RIL2_106	B	5.4
	P593_RIL2_187	B	8.2	P593_RIL2_267	B	5.3
	P593_RIL2_210	B	8.2	P593_RIL2_159	B	5.2

^a^ P593_RIL1, a bi-parental mapping population from a cross between S16-14161 and PI 506593; P593_RIL2, a bi-parental mapping population from a cross between S16-11651 and PI 506593

^b^ Allele, SNP marker position at 41,834,095 bp based on Wm82.a2.v1. Allele A was derived from PI 506593, while allele B was derived from the low-sucrose parental line

^c^ Mean sucrose content across three environments

### Candidate gene identification

**Fig. 1:**
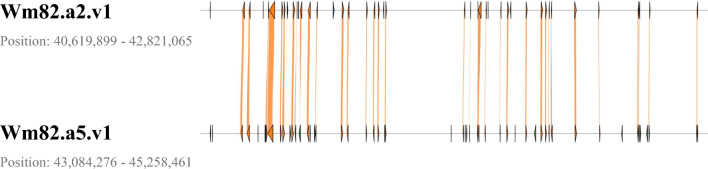
A comparison of the physical position of genes carrying non-synonymous and Indel variants within the major QTL region on Chr. 8 between Wm82.a2.v1 and Wm82.a5.v1 reference genome assemblies. The orange lines indicate the 34 overlapping genes between two reference genome assemblies

Based on the variant identification results on Wm82.a2.v1 and Wm82.a5.v1, the genetic variation and functional annotation of all possible candidate genes within the major QTL region on Chr. 8 were evaluated. Based on Wm82.a2.v1, 175 genes were located in the major QTL region on Chr. 8. Of these, 44 genes carried 85 non-synonymous SNPs and 11 Indels that were only present in PI 506593 (Suppl. Tables S2 and S3). Within the corresponding region of Wm82.a5.v1, 54 genes carried 132 non-synonymous SNPs and 31 Indels in PI 506593 (Suppl. Tables S4 and S5). Among them, 34 genes were common but showed slightly different variants between the two reference genomes (Fig. 1; Table 5). An additional 20 genes on Wm82.a5.v1 were also described in Table 6. The further analyses were conducted based on the 54 candidate genes identified in Wm82.a5.v1. Functional annotations of 54 candidate genes were obtained from Soybase (https://www.soybase.org/) (Tables 5 and 6). According to the BLAST results, the 11 unannotated candidate genes (genes without annotation information in Tables 5 and 6) were described as uncharacterized, hypothetical, unnamed, or missing proteins in soybean (*Glycine max* and *Glycine soja*), common bean (*Phaseolus vulgaris*), and Arabidopsis (*Arabidopsis thaliana*) (Suppl. Table S6).

**Table 5:** The 34 overlapping candidate genes that carried novel variants in the major QTL on Chr. 8 in PI 506593 on Wm82.a2.v1 and Wm82.a5.v1 reference genomes

Wm82.a2.v1			Wm82.a5.v1			Annotation^b^
Gene ID	Position	Variant type^a^	Gene ID	Position	Variant type	
*Glyma.08G293000*	40,766,950–40,775,274	SNP	*Gm_Wm82_23206*	43,220,985–43,228,708	SNP	Sin3 family co-repressor
*Glyma.08G293100*	40,795,741–40,800,913	SNP	*Gm_Wm82_23209*	43,249,676–43,259,978	SNP	Sin3 family co-repressor
*Glyma.08G293800*	40,883,195–40,885,068	Indel	*Gm_Wm82_23219*	43,333,545–43,338,512	Indel	Proline-rich protein 4-like
*Glyma.08G293900*	40,887,462–40,910,754	SNP	*Gm_Wm82_23220*	43,341,530–43,364,303	SNP	Phospholipid-transporting ATPase-like protein
*Glyma.08G294100*	40,942,975–40,947,622	SNP	*Gm_Wm82_23222*	43,396,901–43,401,240	SNP	-^c^
*Glyma.08G294300*	40,953,540–40,957,782	SNP	*Gm_Wm82_23224*	43,407,555–43,415,567	SNP	Galactosyltransferase family protein
*Glyma.08G294800*	40,993,962–41,001,629	SNP	*Gm_Wm82_23227*	43,447,708–43,455,285	SNP	Actin cross-linking protein
*Glyma.08G295100*	41,011537–41,012,029	SNP	*Gm_Wm82_23229*	43,462,185–43,465,739	SNP	Disease resistance protein (TIR-NBS-LRR class)
*Glyma.08G295400*	41,026,575–41,033,381	SNP/Indel	*Gm_Wm82_23231*	43,479,691–43,485,976	SNP/Indel	Disease resistance protein (TIR-NBS-LRR class)
*Glyma.08G295700*	41,062,905–41,071,504	SNP	*Gm_Wm82_23234*	43,516,488–43,524,479	SNP	AMP deaminase-like
*Glyma.08G295800*	41,100,889–41,103,542	SNP	*Gm_Wm82_23237*	43,554,115–43,556,053	SNP	Homeobox associated leucine zipper
*Glyma.08G296300*	41,215,611–41,223,730	SNP	*Gm_Wm82_23243*	43,668,712–43,675,770	SNP	Plant/MEB5-like protein
*Glyma.08G296500*	41,241,857–41,246,757	SNP	*Gm_Wm82_23245*	43,694,878–43,699,270	SNP	Mediator of RNA polymerase II transcription subunit 10
*Glyma.08G297000*	41,326,371–41,329,183	SNP	*Gm_Wm82_23251*	43,779,198–43,781,761	SNP	AP2-like ethylene-responsive transcription factor
*Glyma.08G297400*	41,358,747–41,363,041	SNP	*Gm_Wm82_23255*	43,811,714–43,815,086	SNP	Protein kinase superfamily protein
*Glyma.08G297500*	41,378,414–41,383,832	SNP	*Gm_Wm82_23256*	43,831,982–43,836,271	SNP	Protein IQ-DOMAIN 31-like isoform X6
*Glyma.08G297700*	41,412,445–41,415,775	SNP	*Gm_Wm82_23258*	43,864,913–43,868,558	SNP	F-box/RNI-like superfamily protein
*Glyma.08G299500*	41,767,501–41,769,958	SNP	*Gm_Wm82_23276*	44,211,989–44,214,205	SNP	Peroxisomal adenine nucleotide carrier 1
*Glyma.08G299800*	41,795,912–41,796,546	SNP/Indel	*Gm_Wm82_23279*	44,240,488–44,241,228	SNP	Chitinase A
*Glyma.08G300400*	41,830,891–41,843,406	SNP	*Gm_Wm82_23285*	44,278,415–44,283,458	SNP	–
*Glyma.08G300500*	41,865,484–41,867,085	SNP	*Gm_Wm82_23287*	44,310,063–44,311,664	SNP	Phytochrome kinase substrate 1
*Glyma.08G301300*	41,931,888–41,933,603	SNP	*Gm_Wm82_23293*	44,376,527–44,377,790	SNP	Ribosomal protein L34e
*Glyma.08G301700*	41,962,541–41,968,109	SNP/Indel	*Gm_Wm82_23298*	44,407,335–44,411,704	Indel	–
*Glyma.08G302400*	42,046,386–42,051,525	SNP	*Gm_Wm82_23302*	44,491,765–44,496,137	SNP	Pre-mRNA-splicing factor cwc22
*Glyma.08G303000*	42,114,246–42,121,684	SNP/Indel	*Gm_Wm82_23307*	44,559,451–44,566,133	SNP/Indel	DEAD-box ATP-dependent RNA helicase-like protein
*Glyma.08G303300*	42,134,782–42,139,026	Indel	*Gm_Wm82_23310*	44,579,782–44,584,026	Indel	–
*Glyma.08G303500*	42,150,074–42,151,723	SNP/Indel	*Gm_Wm82_23312*	44,595,014–44,596,723	SNP/Indel	Disease resistance protein (TIR-NBS-LRR class)
*Glyma.08G303600*	42,160,184–42,161,884	SNP	*Gm_Wm82_23313*	44,605,184–44,610,595	SNP	Disease resistance protein (TIR-NBS-LRR class)
*Glyma.08G304300*	42,265,594–42,273,748	Indel	*Gm_Wm82_23320*	44,711,920–44,718,967	Indel	WD repeat-containing protein 26-like
*Glyma.08G305600*	42,374,266–42,376,352	SNP	*Gm_Wm82_23334*	44,821,303–44,823,057	SNP	Disease resistance protein (TIR-NBS-LRR class)
*Glyma.08G307300*	42,551,411–42,553,771	SNP	*Gm_Wm82_23351*	44,989,964–44,992,324	SNP	Receptor serine/threonine kinase
*Glyma.08G307400*	42,556,212–42,559,048	SNP	*Gm_Wm82_23352*	44,994,964–44,997,445	SNP/Indel	Receptor-like protein kinase 1
*Glyma.08G307700*	42,603,547–42,605,822	SNP	*Gm_Wm82_23357*	45,040,796–45,043,149	SNP	60S ribosomal protein L18A-1
*Glyma.08G309200*	42,818,069–42,821,065	SNP	*Gm_Wm82_23372*	45,255,743–45,258,461	SNP	Glycerol-3-phosphate acyltransferase 1

^a^ SNP, Single nucleotide polymorphism; Indel, Insertion and deletion

^b^ Annotation, the annotation was based on Wm82.a5.v1 reference genome using Soybase (https://www.soybase.org/)

^c^ -, Data not available

**Table 6:** Additional candidate genes that carried novel variants in the major QTL on Chr. 8 in PI 506593 on Wm82.a5.v1 reference genomes only

Gene ID	Position	Variant type^a^	Gene ID_Wm82.a2.v1^b^	Annotation^c^
*Gm_Wm82_23199*	43,084,276–43,087,170	SNP	-^d^	–
*Gm_Wm82_23200*	43,094,522–43,095,517	SNP	–	–
*Gm_Wm82_23213*	43,297,226–43,298,074	SNP	–	–
*Gm_Wm82_23216*	43,328,678–43,328,950	SNP	–	–
*Gm_Wm82_23218*	43,331,428–43,333,293	SNP	–	–
*Gm_Wm82_23226*	43,439,054–43,442,901	SNP	*Glyma.08G294700*	Dentin sialophosphoprotein-like isoform X1
*Gm_Wm82_23230*	43,479,691–43,485,976	SNP	–	Disease resistance protein (TIR-NBS-LRR class)
*Gm_Wm82_23235*	43,529,584–43,530,663	SNP	–	Putative nuclease HARBI1-like
*Gm_Wm82_23236*	43,548,217–43,552,351	SNP	–	Putative nuclease HARBI1-like
*Gm_Wm82_23257*	43,861,288–43,861,777	SNP	–	Flocculation protein FLO11-like isoform X4
*Gm_Wm82_23273*	44,158,625–44,159,176	Indel	*Glyma.08G299000*	–
*Gm_Wm82_23277*	44,220,684–44,221,889	SNP/Indel	*Glyma.08G299600*	Probable membrane-associated kinase regulator 6-like
*Gm_Wm82_23278*	44,227,342–44,229,272	SNP	*Glyma.08G299700*	Chitinase A
*Gm_Wm82_23284*	44,276,125–44,276,906	SNP	*Glyma.08G300300*	Chitinase A
*Gm_Wm82_23299*	44,444,016–44,444,536	SNP	–	Protein kinase superfamily protein
*Gm_Wm82_23305*	44,530,279–44,536,400	SNP/Indel	*Glyma.08G302800*	Chromodomain helicase DNA-binding protein
*Gm_Wm82_23345*	44,919,545–44,922,716	SNP	*Glyma.08G306700*	Disease resistance protein (TIR-NBS-LRR class)
*Gm_Wm82_23353*	45,001,129–45,004,003	SNP	–	Phototropin 1
*Gm_Wm82_23356*	45,029,632–45,035,122	SNP	–	Receptor-like protein 12-like
*Gm_Wm82_23371*	45,253,027–45,253,530	Indel	–	–

^a^ SNP, Single nucleotide polymorphism; Indel, Insertion and deletion

^b^ Gene ID_Wm82.a2.v1, the corresponding gene ID based on Wm82.a2.v1

^c^ Annotation, the annotation was based on Wm82.a5.v1 reference genome using Soybase (https://www.soybase.org/)

^d^ -, Data not available

**Fig. 2:**
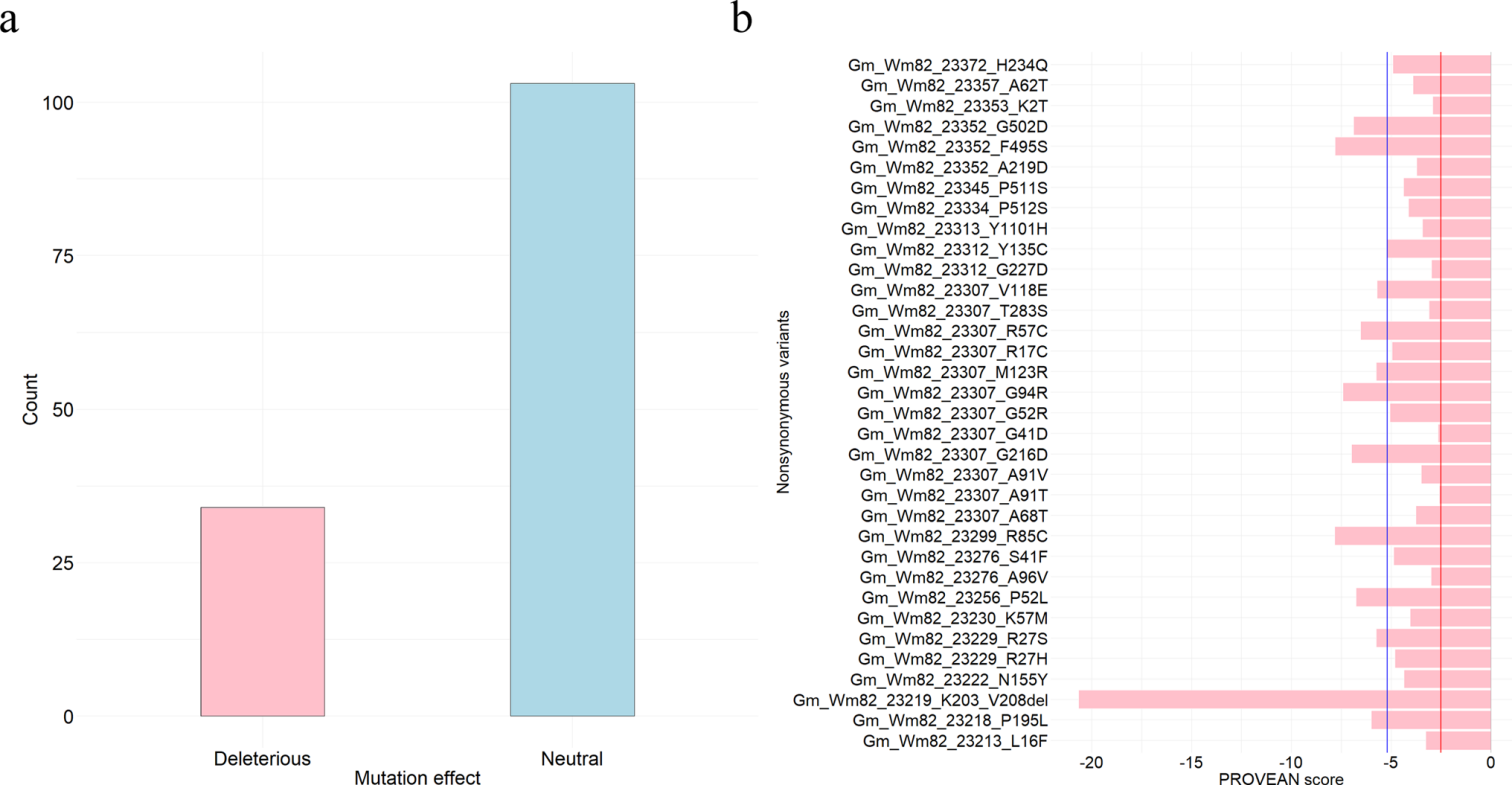
Results from PROVEAN analysis testing 132 non-synonymous SNP and five non-frameshift Indel variants within the major QTL region on Chr. 8 showed **a** a total number of deleterious and neutral variants and **b** PROVEAN scores of deleterious variants. The blue line indicates the average PROVEAN score of deleterious variants, which was − 5.288. The red line indicates the significant threshold, which was − 2.5

A total of 132 non-synonymous SNPs and five non-frameshift Indels in the major QTL region on Chr. 8 were evaluated for their biological impacts on the candidate gene functions using PROVEAN (Fig. 2). Based on the suggested threshold (PROVEAN score ≤ − 2.5), the donor parent, PI 506593, carried 34 deleterious variants, which can significantly affect the biological functions of 18 candidate genes (Fig. 2a). Among these, the deletion of six amino acids from Lysine (K) 203 to Valine (V) 208 (PROVEAN score = − 20.674) in *Gm_Wm82_23219* (*Glyma.08G293800*, Wm82.a2.v1) showed the most deleterious impact on the biological function (Fig. 2b). One amino acid substitution from Arginine (R) to Cysteine (C) at position 85 (PROVEAN score = − 7.845) in *Gm_Wm82_23299* (Gene name was not available on Wm82.a2.v1) was the most deleterious non-synonymous SNP variant, followed by the amino acid substitution from Phenylalanine (F) to Serine (S) at position 495 (PROVEAN score = − 7.822) in *Gm_Wm82_23352* (*Glyma.08G307400*, Wm82.a2.v1) in PI 506593. The average PROVEAN score of those deleterious variants was -5.288 (Fig. 2b).

**Fig. 3:**
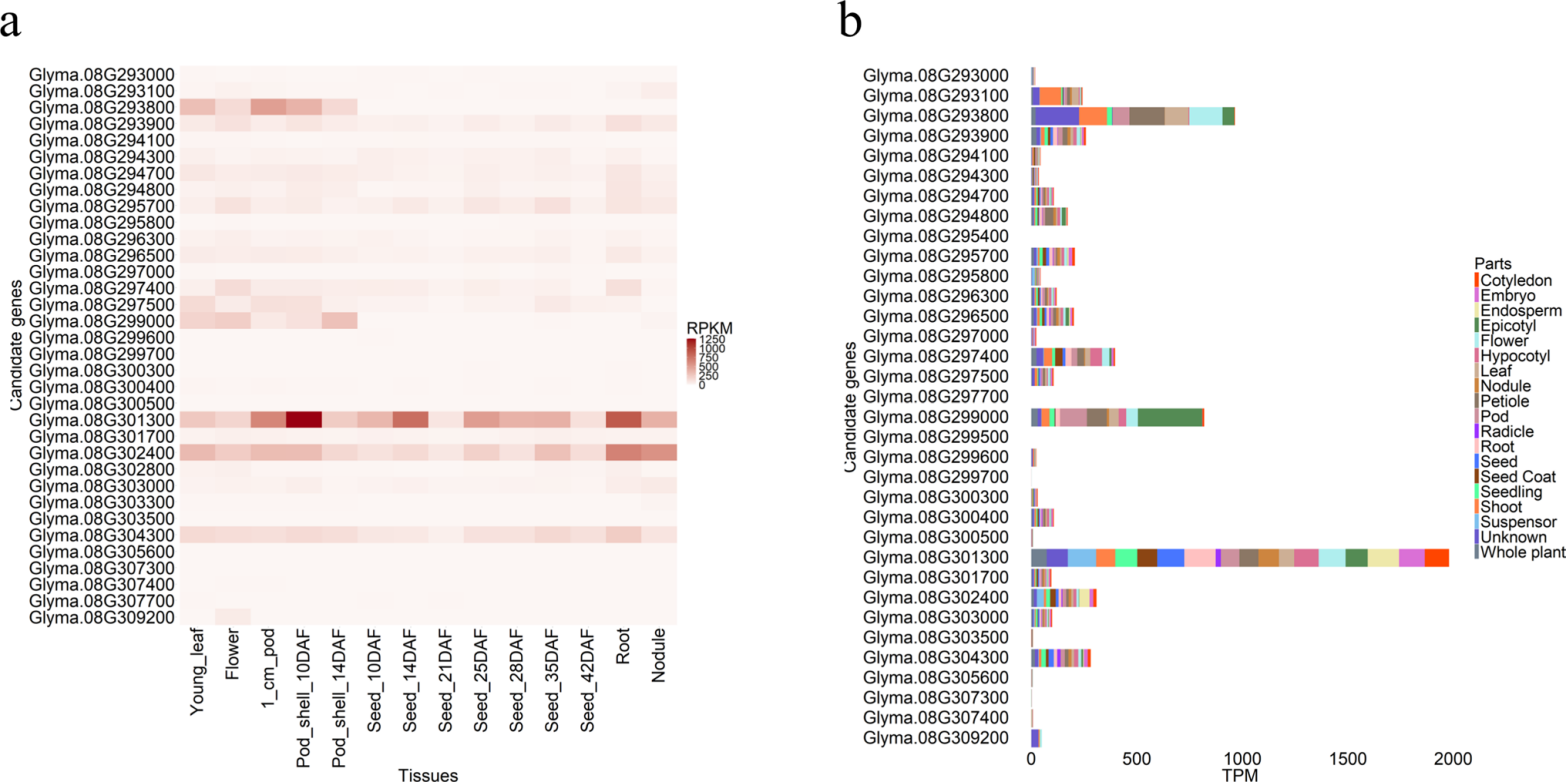
The candidate gene expression levels within the major QTL region on Chr. 8 using publicly available gene expression datasets from **a** Severin et al. (2010) and **b** Almeida-Silva et al. (2023), respectively. The read counts for **a** and **b** were normalized using Reads Per Kilobase of transcript per Million mapped reads (RPKM) and Transcripts Per Million (TPM) method, respectively

RNA-seq analysis utilized only genes with corresponding gene IDs for either Wm82.a1.v1 or Wm82.a4.v1 required for RNA-seq data search in Severin et al. (2010) and Almeida-Silva et al. (2023), respectively. The corresponding gene IDs between Wm82.a1.v1, Wm82.a2.v1, Wm82.a4.v1, and Wm82.a5.v1 were described in Suppl. Table S7. A total of 34 candidate genes were evaluated for their expression levels in different plant tissues using publicly available databases described by Severin et al. (2010) and Almeida-Silva et al. (2023), respectively (Fig. 3). According to the RNA-seq dataset from Severin et al. (2010), *Glyma.08G301300* (*Gm_Wm82_23293*, Wm82.a5.v1), *Glyma.08G302400* (*Gm_Wm82_23302*, Wm82.a5.v1), and *Glyma.08G304300* (*Gm_Wm82_23320*, Wm82.a5.v1) showed noticeably higher expression levels in seed tissues during seed development stages than other candidate genes (Fig. 3a). Two genes, *Glyma.08G293800* (*Gm_Wm82_23219*, Wm82.a5.v1) and *Glyma.08G299000* (*Gm_Wm82_23273*, Wm82.a5.v1), showed higher gene expression levels during early pod development stages (from 1_cm_pod to Pod_shell_14DAF). According to the RNA-seq dataset from Almeida-Silva et al. (2023), *Glyma.08G293800* and *Glyma.08G301300* showed outstanding gene expression levels across 19 plant parts (> 1000 TPM) (Fig. 3b). The high gene expression levels of *Glyma.08G293800* were observed in the shoot, leaf, flower, and pod, while *Glyma.08G301300* was highly expressed in all plant parts.

## Discussion

Similar to other seed composition traits in soybean, sucrose content fluctuates in response to various environmental factors, such as temperature, precipitation, and air and soil humidity, that affect plant growth, flowering time, and seed development (Jo et al. 2018; Lee et al. 2023a, b; Tarumingkeng and Coto 2003; Wang et al. 2023a). In this study, the average sucrose content was the highest in BREC_22 and the lowest in FDREEC_21 (Table 1). Although many studies reported a negative correlation between temperature and sucrose content (lower temperature induces higher sucrose) during pod-filling stages, our study did not strictly follow this trend. However, according to meteorological data from nearby weather stations (https://www.wunderground.com/), from late September to October, BREC_22 had the lowest average temperature of 15.2 °C, while FDREEC_21 had the highest average temperature of 17.8 °C. This observation suggests that the sucrose content of soybean may be more sensitive to lower temperatures during the late reproductive stages (R6–R7) than during the early pod-filling stages (R3–R5). Soil moisture is another important environmental factor affecting the sucrose content. However, the relationship between soil moisture and sucrose content at present is unclear (Du et al. 2020; Wang et al. 2021; Wijewardana et al. 2019). We used the dew point temperature to determine the correlation between soil moisture and sucrose content. The dew point temperature provides an actual amount of water vapor in the air and positively correlates with soil moisture content (Leelamanie 2010). In this study, the sucrose content was negatively correlated with the dew point temperature during the pod-filling stages, indicating that the lower the soil moisture content, the higher the sucrose content. This finding was consistent with previous studies showing the negative correlation between soil moisture and sucrose content in soybean (Du et al. 2020; Wang et al. 2023a).

A total of 11 QTLs on Chrs. 4, 5, 6, 8, 10, and 13 were identified in two populations sharing a high sucrose donor parent across three environments (Table 3). Among them, QTLs on Chr. 8 (*qSUC_08.1*, *qSUC_08.2*, *qSUC_08.3*, and *qSUC_08.4*) were consistently detected across all environments in two populations except BREC_22 in P593_RIL1 (Table 3). Since two populations shared the same donor parent, a genomic region harboring these QTLs on Chr. 8 was identified as a major QTL region (the physical interval of 40,597,410–42,861,364 bp, Wm82.a2.v1) that underlies the high sucrose trait in PI 506593. In a previous study, this major QTL region was located near the QTL (43,602,528–43,670,249 bp) identified by Lee et al. (2016) from a population derived from a cross of Keunolkong × Iksan 10, although they did not overlap with our findings. Other minor QTLs identified on Chrs. 4, 5, 6, 10, and 13 in our study seemed unstable and inconsistent across environments (Table 3). Two QTLs, *qSUC_06.1* and *qSUC_06.2*, were only identified on Chr. 6 in the P593_RIL1 at the physical intervals of 16,133,204–21,786,787 bp and 21,786,787–46,805,251 bp, respectively (Table 3). However, due to the lack of polymorphic markers on Chr. 6, the two QTLs constituted more than 50% of the entire chromosome, which spans approximately 30 Mbp. In other studies, most of the sucrose-related QTLs identified on Chr. 6 were located within this region (Liu et al. 2023; Patil et al. 2018; Skoneczka et al. 2009). A minor QTL on Chr. 13 (*qSUC_13.1*) identified in P593_RIL1 was located at the physical interval of 25,268,844–25,927,261 bp, which was close to the QTL that Maughan et al. (2000) previously identified at the physical interval of 26,196,486–28,912,864 bp (‘Sucrose 1–5’ in Soybase). The other minor QTLs on Chrs. 4, 5, 10, and 13 have not been reported near these regions from previous studies.

Desirable marker testing in both populations showed that the SNP marker positioning at 41,834,095 bp on Chr. 8 had a high efficiency in differentiating between phenotypically extreme groups in P593_RIL1. On the other hand, the SNP marker had a high discrimination power for only low sucrose lines in P593_RIL2. This discrepancy could occur due to the lack of SNP markers anchored on Chr. 8 in P593_RIL2. P593_RIL1 contained 104 SNP markers with an average interval of 1.4 cM, while P593_RIL2 contained only 24 SNP markers with an average interval of 4.9 cM on Chr. 8 (Table 2). Although the significant SNP marker identified in this study could be included in the marker-assisted selection pipeline for high sucrose soybean breeding along with other molecular markers, such as *rs2W331-*, *rs2T107I*, *rs3snp5*/*rs3snp6*, *MIPS1,* further investigations, such as fine mapping followed by molecular marker development, within the major QTL region, will increase the marker efficiency and precision.

In this study, the WGS data of the three parental lines, PI 506593, S16-14161, and S16-11651, constituting the two mapping populations, were analyzed to identify SNP and Indel variants within the significant QTL regions. This genomic information enabled us to narrow down the candidate genes within sucrose-related QTL regions identified by a conventional bi-parental mapping analysis. Furthermore, this study was the first to employ the high-quality reference genome assembly of Wm82.a5.v1 in QTL analysis compared to the most widely used reference genome version of Wm82.a2.v1. To date, most novel genomic findings in soybean research were identified and reported based on Wm82.a2.v1. However, some variants identified in Wm82.a2.v1 were not found in Wm82.a5.v1 and vice versa (Fig. 1; Suppl. Tables S4 and S5). This is mainly because most of the gaps and scaffold breaks in Wm82.a2.v1 were completed in Wm82.a5.v1, resulting in different numbers of total genes between Wm82.a2.v1 (56,044 genes) and Wm82.a5.v1 (58,287 genes). Specifically, the total length of Chr. 8 in Wm82.a5.v1 is 50,302,612 bp, whereas that of Chr. 8 in Wm82.a2.v1 is 47,837,940 bp.

We studied potential candidate genes associated with sucrose content in PI 506593 using PROVEAN and RNA-seq analysis. These analyses were informative in identifying promising candidate genes among the 54 genes within the major QTL region. However, further investigations, such as fine mapping, molecular marker development, and gene editing, will be required to pinpoint causal variants and genes. Three candidate genes, *Gm_Wm82_23219* (*Glyma.08G293800*, Wm82.a2.v1), *Gm_Wm82_23299* (Gene name was not available on Wm82.a2.v1), and *Gm_Wm82_23352* (*Glyma.08G307400*, Wm82.a2.v1) were the most significant in PROVEAN (Fig. 2). Interestingly, the functional annotations of these candidate genes are proline-rich protein 4-like, protein kinase superfamily protein, and receptor-like protein kinase 1, respectively, which are responsible for the stress reaction against various abiotic stressors, such as drought, salinity, etc. (Cui et al. 2022; Kavi Kishor et al. 2015; Rajasheker et al. 2022) (Tables 5 and 6). Especially during the reproductive process, those genes play a key role in the onset of leaf senescence, which is crucial in disassembling macromolecules and reallocating nutrients into developing seeds (Yang et al. 2022). Sucrose accumulation begins at the early pod-filling stages and ceases when the seed color turns yellow, which is the onset of leaf senescence (Obendorf et al. 1997). Thus, the candidate genes could be responsible for the stress-induced leaf senescence and regulation of the soluble carbohydrate metabolic process (Yang et al. 2022). Also, a recent study identified a stable major QTL associated with sucrose content in peanut (*Arachis hypogaea* L.) and highlighted a candidate gene encoding receptor-like protein kinase (Li et al. 2023). We also identified six additional candidate genes, including *Gm_Wm82_23229* (*Glyma.08G295100*, Wm82.a2.v1), *Gm_Wm82_23230* (Gene name was not available on Wm82.a2.v1), *Gm_Wm82_23312* (*Glyma.08G303500*, Wm82.a2.v1), *Gm_Wm82_23313* (*Glyma.08G303600*, Wm82.a2.v1), *Gm_Wm82_23334* (*Glyma.08G305600*, Wm82.a2.v1), and *Gm_Wm82_23345* (*Glyma.08G306700*, Wm82.a2.v1), that were annotated as disease resistance protein (Tables 5 and 6). As a main signaling molecule, the translocation and utilization of sucrose are significantly affected by not only external environmental stressors but also biotic stressors (Aluko et al. 2021; Tauzin and Giardina 2014). Therefore, the variants significantly affecting the biological function of stress response-related genes could regulate the reallocation of sucrose, resulting in the modification of seed sucrose content in soybean.

Furthermore, a subsequent RNA-seq analysis highlighted five other candidate genes. They were *Glyma.08G293800* (*Gm_Wm82_23219*, Wm82.a5.v1), *Glyma.08G299000* (*Gm_Wm82_23273*, Wm82.a5.v1), *Glyma.08G301300* (*Gm_Wm82_23293*, Wm82.a5.v1), *Glyma.08G302400* (*Gm_Wm82_23302*, Wm82.a5.v1), and *Glyma.08G304300* (*Gm_Wm82_23320*, Wm82.a5.v1). Of these, *Glyma.08G293800,* highlighted from PROVEAN results, also showed high gene expression levels in pod tissues (Fig. 3a). Since seed composition traits are closely related to reproductive stages, the identification of candidate genes with high gene expression levels in the pod and seed tissues is important (Lee et al. 2023b). Two genes, *Glyma.08G301300* and *Glyma.08G302400*, were related to the post-transcriptional process (Ribosomal protein L34e and Pre-mRNA-splicing factor cwc22, respectively), but further investigation will be required to confirm the association with sucrose-related genes. The annotation of *Glyma.08G299000* was unknown, uncharacterized, and unnamed in *Glycine max*, *Glycine soja*, *Phaseolus vulgaris*, and *Arabidopsis thaliana*, respectively, based on BLAST results, although it showed relatively high gene expression levels in pod tissues during early reproductive stages (Fig. 3a).

Despite not being highlighted in PROVEAN and RNA-seq analysis, a novel variant in one candidate gene, *Gm_Wm82_23224* (*Glyma.08G294300,* Wm82.a2.v1), which is closely related to sucrose accumulation, was identified in PI 506593 on both reference genomes. *Gm_Wm82_23224* encodes a galactosyltransferase family protein, of which two well-known soluble carbohydrate-related genes, RS2 and RS3, also encode similar proteins, galactinol-sucrose galactosyltransferase (https://www.soybase.org/). Also, *Gm_Wm82_23224* is homologous to the *GATL4–6* genes, sharing 58–60% amino acid similarities in *Arabidopsis thaliana*. The *GALT* genes encode the galactosyltransferase family protein in *Arabidopsis thaliana*. Galactosyltransferase plays a key role in transferring galactose and regulating carbon partitioning between sucrose and raffinose in soybean seeds (Saravitz et al. 1987; Singer et al. 2023). Although this gene was not highlighted in PROVEAN and RNA-seq analysis, the variant is still worth highlighting due to the biological function of the gene directly related to sucrose accumulation.

## Conclusion

In this study, QTL analyses using two mapping populations derived from the same donor parent, PI 506593, identified a major QTL region on Chr. 8 controlling sucrose content in soybean seeds. The WGS data of the parental lines aligned to Wm82.a2.v1 and Wm82.a5.v1 were used to identify SNP and Indel variants within the major QTL region and candidate genes. Based on PROVEAN and RNA-seq analysis, 18 and 5 candidate genes were highlighted, respectively. Among these, *Gm_Wm82_23219* (*Glyma.08G293800*, Wm82.a2.v1) in PI 506593 carried the most deleterious non-synonymous variant and was highly expressed in pod tissues during the early reproductive stages in soybean. The findings in our study suggested that candidate genes and novel variants within the major QTL region on Chr. 8 can be useful genetic resources for improving carbohydrate profiles in new soybean cultivars aimed at animal feed efficiency and human consumption.

## Supplementary Information

Below is the link to the electronic supplementary material.

Supplementary file1 (XLSX 59 KB)

## Data Availability

The datasets generated during and/or analyzed during the current study are available from the corresponding author upon reasonable request.
